# Population Characteristics and Organ Procurement Organization Performance Metrics

**DOI:** 10.1001/jamanetworkopen.2023.36749

**Published:** 2023-10-03

**Authors:** Rocio Lopez, Sumit Mohan, Jesse D. Schold

**Affiliations:** 1 Division of Transplant Surgery, Department of Surgery, University of Colorado Anschutz Medical Campus, Aurora; 2 Quantitative Health Sciences, Lerner Research Institute, Cleveland Clinic, Cleveland, Ohio; 3 Division of Nephrology, Department of Medicine, Vagelos College of Physicians and Surgeons, Columbia University, New York, New York; 4 Department of Epidemiology, Mailman School of Public Health, Columbia University, New York, New York; 5 Department of Epidemiology, University of Colorado Anschutz Medical Campus, Aurora

## Abstract

**Importance:**

In 2020, the Centers for Medicare & Medicaid Services (CMS) issued a final rule updating the Organ Procurement Organization (OPO) Conditions for Coverage. This rule evaluates OPO performance based on an unadjusted donation rate and an age-adjusted transplant rate; however, neither considers other underlying population differences.

**Objective:**

To evaluate whether adjusting for age and/or area deprivation index yields the same tier assignments as the cause, age, and location consistent (CALC) tier used by CMS.

**Design, Setting, and Participants:**

This retrospective cross-sectional study examined the performance of 58 OPOs from 2018 to 2020 across the entire US. A total of 12 041 778 death records were examined from the 2017 to 2020 National Center for Health Statistics’ Restricted Vital Statistics Detailed Multiple Cause of Death files; 399 530 of these met the definition of potential deceased donor. Information about 42 572 solid organ donors from the Scientific Registry of Transplant Recipients was also used. Statistical analysis was performed from January 2017 to December 2020.

**Exposure:**

Area deprivation of donation service areas and age of potential donors.

**Main Outcome and Measures:**

OPO performance as measured by donation and transplant rates.

**Results:**

A total of 399 530 potential deceased donors and 42 572 actual solid donor organs were assigned to 1 of 58 OPOs. Age and ADI adjustment resulted in 19.0% (11 of 58) to 31.0% (18 of 58) reclassification of tier ratings for the OPOs, with 46.6% of OPOs (27 of 58) changing tier ranking at least once during the 3-year period. Between 6.9% (4 of 58) and 12.1% (7 of 58) moved into tier 1 and up to 8.6% (5 of 58) moved into tier 3.

**Conclusions and Relevance:**

This cross-sectional study of population characteristics and OPO performance metrics found that adjusting for area deprivation and age significantly changed OPO measured performance and tier classifications. These findings suggest that underlying population characteristics may alter processes of care and characterize donation and transplant rates independent of OPO performance. Risk adjustment accounting for population characteristics warrants consideration in prospective policy and further evaluation of quality metrics.

## Key Points

**Question:**

Does adjusting for decedent’s age and/or area deprivation index yield the same performance tier assignments as the tier used by the Centers for Medicare & Medicaid Services?

**Findings:**

In this cross-sectional study that evaluated 58 organ procurement organizations from 2018 to 2020, adjustment for area deprivation index (ADI) resulted in 15.5% to 27.6% tier reclassification based on ADI alone and 19.0% to 31.0% based on age and ADI each year.

**Meaning:**

These results suggest that underlying population characteristics may alter processes of care and characterize donation and transplant rates independent of performance. Risk adjustment accounting for population characteristics warrants consideration in prospective policy and further evaluation of quality metrics.

## Teaser

This cross-sectional study investigates whether adjusting for age and/or area deprivation index yields the same tier assignments for organ procurement organizations as the cause, age, and location consistent tier used by Centers for Medicare & Medicaid Services.

## Introduction

In December 2020, the Centers for Medicare & Medicaid Services (CMS) published an amended regulation updating the Organ Procurement Organization (OPO) Conditions for Coverage.^1^ This rule uses the cause, age, and location consistent (CALC) organ donation method and defines donor potential as the number of inpatient deaths among patients aged 75 years or younger with a primary cause of death consistent with organ donation. Donation and transplant rates are defined as the number of organ donors and organs transplanted, respectively, as percentages of the donor potential.^1^ The donation rate is unadjusted, and the transplant rate is risk adjusted for age of donor potential. CMS deemed the age cutoff of 75 years or younger used to define donor potential as sufficient age adjustment for donation and kidney transplantation rates.^1^

OPOs are to be evaluated yearly and certified or decertified every 4 years based on a single year’s data starting in 2024. Tier 1 OPOs will be recertified for an additional 4 years, have exclusive rights to their donation service area (DSA), and can compete for any other open DSA. Tier 2 OPOs must compete to keep their DSA. Tier 3 will receive a notice of decertification and will be replaced by a better-performing OPO unless an appeal is filed and won.^1^ Thus, the ramifications of these CMS metrics are critical to OPOs, which have the important responsibility of managing the deceased donor national transplantation system and which directly affect patients with end-stage organ disease requiring a life-saving donor organ transplant.

There is increasing evidence that social risk factors are associated with poor health and worse health outcomes,^2,3,4,5,6^ and the debate over adjusting performance metrics for social risk factors is gaining interest. Social risk adjustment advocates argue that excluding these factors from performance metrics can overly penalize hospitals and centers serving populations with high social risk and adjustment can help address this concern.^7,8,9^ For example, safety-net hospitals were more likely penalized and had larger penalties than non–safety-net hospitals under the initial CMS Health Readmissions Reduction Program (HRRP).^10^ This led to Congress passing the 21st Century Cures Act, which aimed to establish beneficiary equity in the HRRP by evaluating hospitals’ performance relative to peer hospitals with similar proportions of patients dually eligible for Medicare and Medicaid.^11^ In response, the HRRP began to adjust for social risk, which led to substantial shifts in penalties.^12,13,14^

The OPO performance metrics do not consider underlying differences between DSAs yet there is substantial geographic variation in social risk factors.^15,16^ Higher area deprivation index (ADI), as well as concentrated disadvantage, have been shown to be associated with higher comorbidity rates^17^ and higher organ discard rates.^18^ Higher neighborhood disadvantage, lower socioeconomic level, and lower educational attainment have also been found to be associated with lower organ donor registration and authorization rates.^19,20,21^ In addition, both donor consent rates and donation rates are significantly associated with age.^22,23,24^ Our aim was to evaluate whether adjusting metrics for age and/or ADI yields the same tier assignments as the CMS CALC tiers.

## Methods

### Data

This retrospective cross-sectional study uses death data from the Centers for Disease Control and Prevention (CDC), National Center for Health Statistics’ Restricted Vital Statistics Detailed Multiple Cause of Death (MCOD) 2017 to 2020 files. MCOD provides county-level mortality data based on death certificates of US residents.^25,26^ We identified all deaths occurring within the 50 states, the District of Columbia, Puerto Rico, and US Virgin Islands. Deaths were assigned to 1 of 58 active OPOs between 2017 and 2020 based on the county where they occurred. We used the Scientific Registry of Transplant Recipients (SRTR) OPO-specific reports for each corresponding year to determine which counties are assigned to each DSA.^27^

We also used data from SRTR Standard Analysis Files supplied in June 2021. The SRTR includes data on all donors, wait-listed candidates, and transplant recipients in the US submitted by the members of the Organ Procurement and Transplantation Network (OPTN).^28^ The Health Resources and Services Administration, US Department of Health and Human Services, provides oversight to the activities of the OPTN and SRTR contractors. We identified donors and transplanted organs and assigned to 1 OPO based on the hospital county. SRTR and MCOD data were merged at the OPO level.

**Figure 1.**
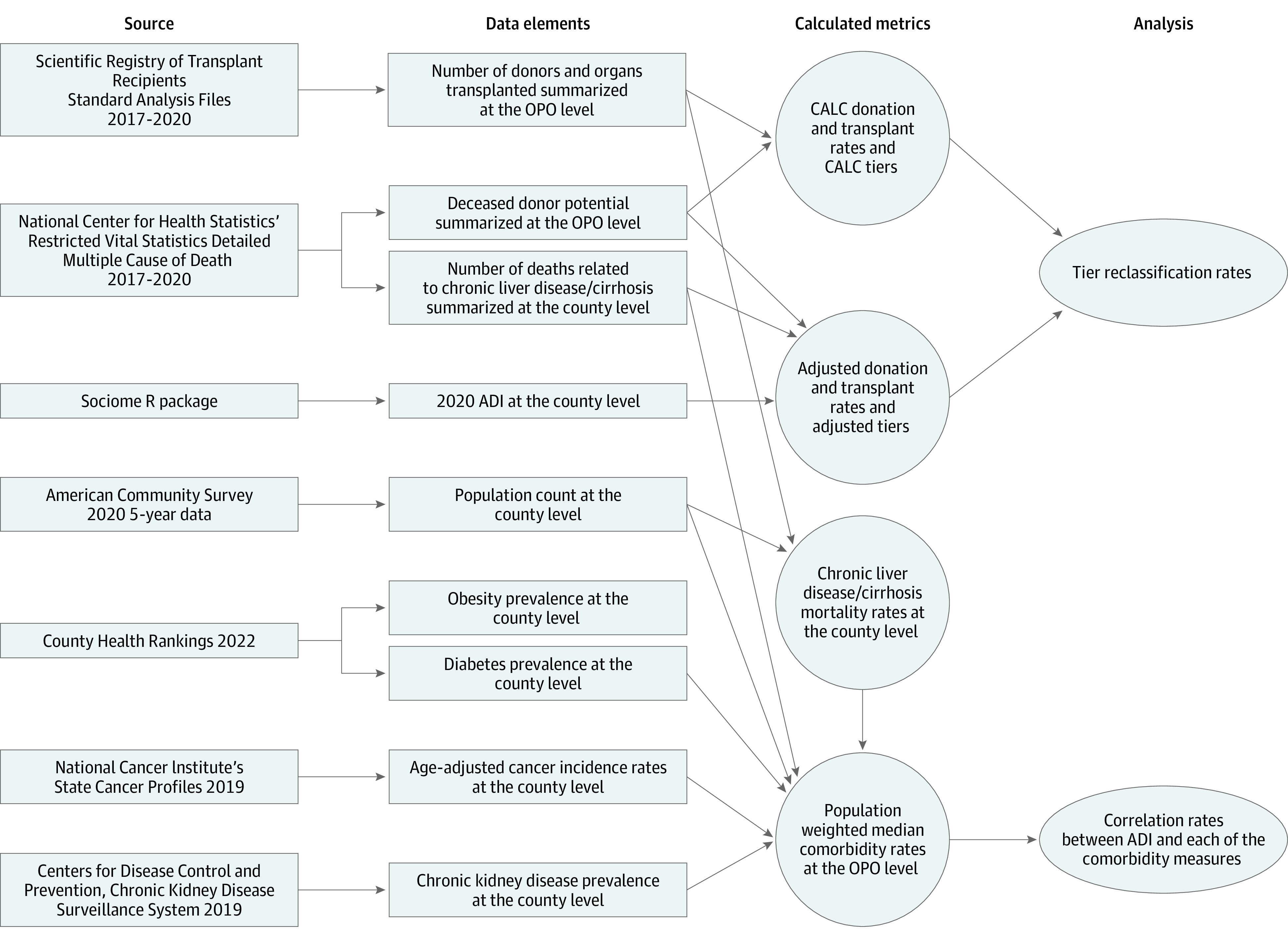
Flowchart of Data Sources and Data Management Process ADI indicates area deprivation index; CALC, cause, age, and location consistent; OPO, Organ Procurement Organization.

We obtained the 2020 ADI at the county level using the sociome R package.^29,30^ This index is calculated using the 2016 to 2020 American Community Survey (ACS) 5-year data and is a variation of the ADI created by the Health Resources & Services Administration^31^ that allows calculating the index at different geographic levels, including the county level. The ADI is computed using 15 measures that fit into 1 of 3 factors: (1) financial strength, (2) economic hardship and inequality, and (3) educational attainment; higher ADI values mean more area disadvantage.^30^ We ranked all counties and determined a national quintile rank for each. Deaths from MCOD and donors or transplanted organs from SRTR were assigned an ADI based on county of residence of the participant. We also obtained county-level data on obesity prevalence, diabetes prevalence, cancer incidence rates, chronic kidney disease (CKD) prevalence, chronic liver disease (CLD) and cirrhosis mortality rates, and population counts as detailed in Figure 1.^26,32,33,34,35^

This study followed the Strengthening the Reporting of Observational Studies in Epidemiology (STROBE) reporting guideline for cross-sectional studies and was approved by the Cleveland Clinic institutional review board. The study was approved as exempt with no informed consent needed given the retrospective analysis of deidentified data.

### Donor Potential, Metrics, and Tier Assignments

Following the 2020 CMS rule, we used the CALC organ donation method to define donor potential.^1^ Comprehensive explanations of definitions, formulas for calculating donation and transplant rates, corresponding 1-sided 95% upper confidence limit, and method for tier assignment can be found in the OPO Annual Public Aggregated Performance Report–User Guide published by CMS Quality, Certification, and Oversight Reports.^36^

### Missing Data

Of 12 041 778 deaths, 25 548 (0.21%) did not include information regarding the participant’s county of residence. Out of 12 016 230 participants for whom we had both place of residence and death, 11 542 796 (96.1%) resided and died within the same DSA. Out of 42 572 donors, 479 (1.13%) did not have information on the donor’s residence. Of the 42 093 who had information on both hospital and residence, 37 606 (89.3%) resided and died within the same DSA. Therefore, when place of residence was not available, we assigned ADI based on the county where the death occurred.

### Statistical Analysis

#### OPO Profiles

ADI, comorbidity measures, and population count were merged at the county level. Population-weighted medians were calculated for each OPO using the 2020 county-DSA assignments from SRTR. In addition, we ranked all 58 OPOs based on population-weighted ADI and divided into quintiles for purposes of visual representation. Spearman correlations were used to assess the association between the various measures at the OPO level.

#### OPO Metrics

Donation, kidney transplant, and organ transplant rates were adjusted to account for differences in age and ADI distribution of each OPO’s potential donor population. We used indirect standardization following the approach used by the 2020 CMS rule for age adjustment.^1,36^ This approach was used for the ADI adjustment as well as age and ADI adjustment (eMethods in Supplement 1).

Each year, we placed OPOs in rank order and determined the median and top 25% thresholds for each raw and adjusted rate; these threshold values were then used for the next year’s tier assignments. OPOs were placed into 1 of 3 tiers in 2018, 2019, and 2020 based on the 1-sided 95% upper limit and the prior year’s threshold values for both the donation and transplant rate as follows: (1) CALC tier: uses raw donation rate and age-adjusted transplant rate (or Legacy of Life Hawaii [HIOP] raw kidney transplant rate); this is the tier used by CMS for certification and decertification decisions^1,36^; (2) age-adjusted tier: uses age-adjusted donation rate and age-adjusted transplant rate (or HIOP age-adjusted kidney transplant rate); (3) ADI-adjusted tier: uses ADI-adjusted donation rate and age and ADI–adjusted transplant rate (or HIOP ADI-adjusted kidney transplant rate); (4) age and ADI–adjusted tier: uses age and ADI–adjusted donation rate and age and ADI–adjusted transplant rate (or HIOP age and ADI–adjusted kidney transplant rate).

In addition, we established separate ranks for donation and transplant rates, which are together used to determine performance tiers, following the same method used for tier assignments. We also compared CALC rates to adjusted rates using Wilcoxon signed-rank tests. Finally, we calculated tier reclassification rates as the percentage of OPOs that change tiers compared with the CALC tier. Statistical tests were performed at a significance level of .05 using SAS software version 9.4 (SAS Institute) from January 2017 to December 2020.

## Results

### OPO Profiles

**Figure 2.**
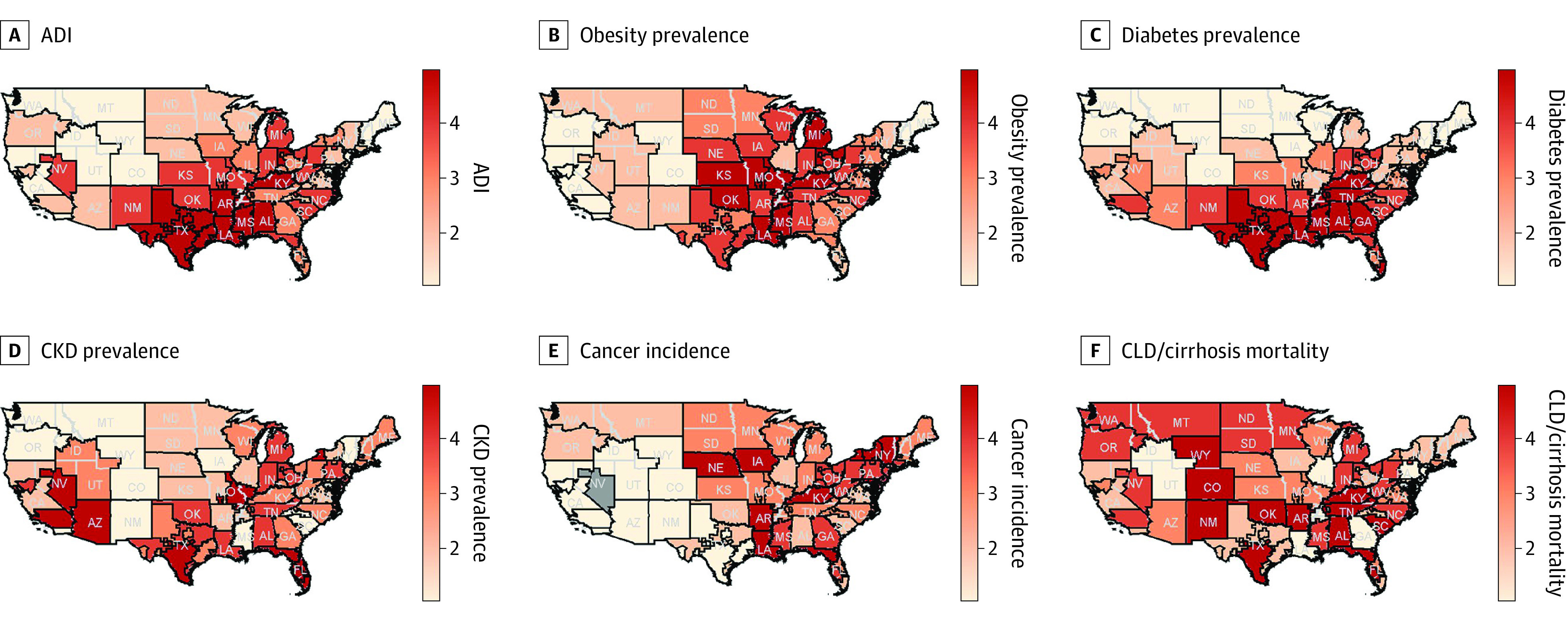
Geographic Variation in Area Deprivation Index (ADI), Obesity Prevalence, Diabetes Prevalence, Chronic Kidney Disease (CKD) Prevalence, Cancer Incidence, and Chronic Liver Disease (CLD)/Cirrhosis Mortality^a^ Prevalence range (1-5) indicates quintiles, with 1 being the lowest quintile and 5 the highest quintile. ^a^Cancer incidence rates are age-adjusted and represent cases per 100 000 persons. Black outlines represent donation service area boundaries, and gray lines denote state boundaries. Gray color indicates no data available.

**Figure 3.**
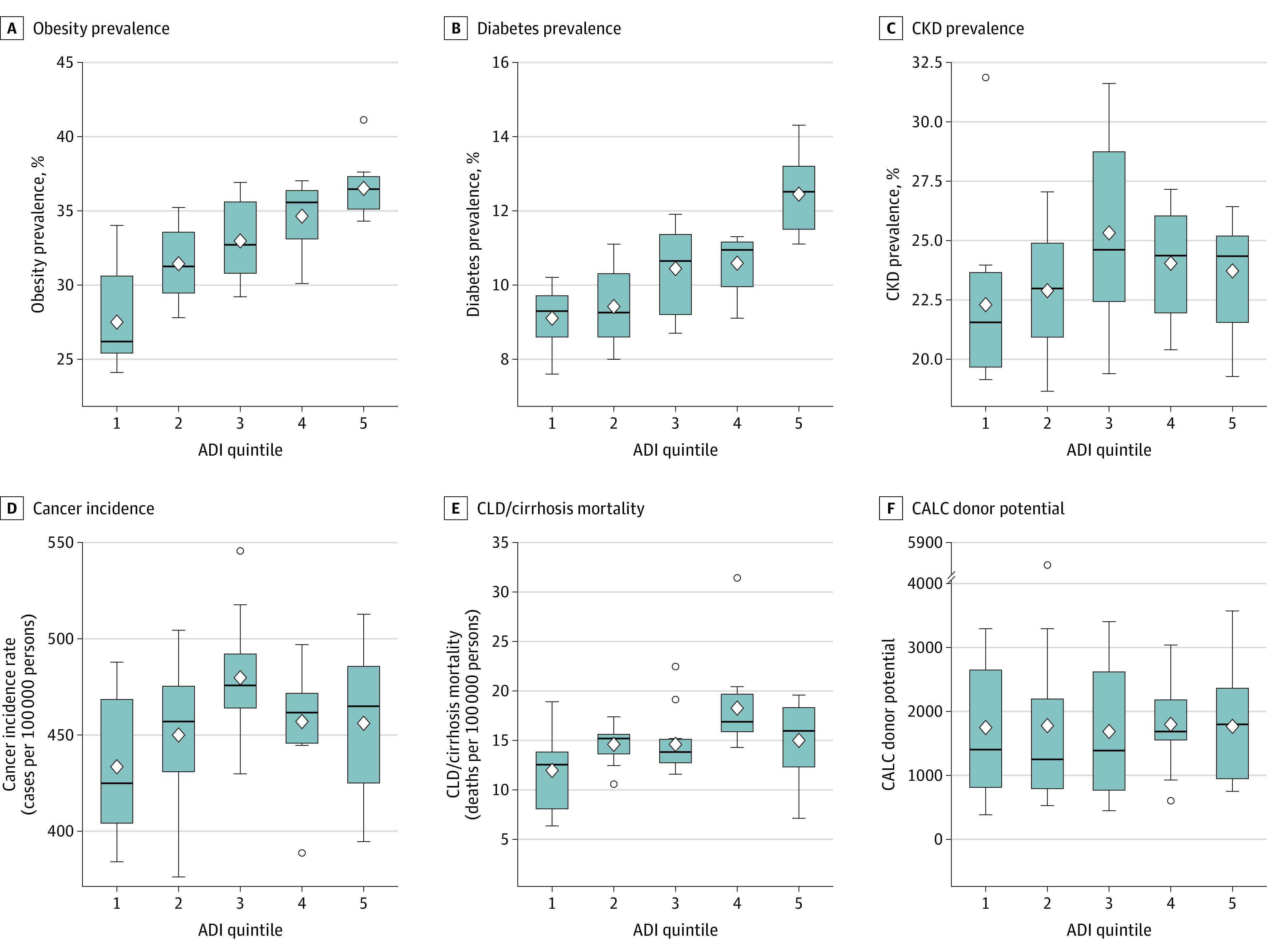
Obesity Prevalence, Diabetes Prevalence, Chronic Kidney Disease (CKD) Prevalence, Cancer Incidence, Chronic Kidney Disease (CLD)/Cirrhosis Mortality, and Cause, Age, and Location Consistent (CALC) Donor Potential by Area Deprivation Index (ADI) Quintile Box is drawn from the first quartile to the third quartile, and the vertical line goes through the box at the median value. The diamond within the box represents the mean value. The whiskers extend to the minimum and maximum observations above or below the lower and upper fences (±1.5 × IQR). Circles indicate outliers (observations that are more extreme than the lower or upper fences).

A total of 399 530 potential deceased donors and 42 572 actual solid donor organs were assigned to 1 of 58 OPOs. ADI; obesity, diabetes, and CKD prevalence; cancer incidence rates; and CLD and cirrhosis mortality rates varied significantly across DSAs (Figure 2). At the OPO level, higher deprivation was strongly correlated with increased obesity (ρ = 0.75; *P* < .001) and diabetes (ρ = 0.72; *P* < .001) rates. There was weak-moderate correlation between higher deprivation and CKD prevalence (ρ = 0.26; *P* = .047), CLD and cirrhosis mortality (ρ = 0.37; *P* = .004), CALC donation rate (ρ = −0.23; *P* = .003), and CALC transplant rate (ρ = −0.23; *P* = .002). In addition, cancer incidence was higher in the top 3 ADI quintiles but not significantly correlated (ρ = 0.22; *P* = .11). Despite these differences, the CALC donor potential was similar across all ADI quintiles with slightly increased values observed at quintiles 4 and 5 (ρ = 0.07; *P* = .62) (Figure 3).

### OPO Metrics

Out of 12 041 778 deaths across the US, 399 530 (3.3%) met the definition of potential deceased donor and were used to calculate performance metrics. All rates and tiers for each OPO are in eTables 1, 2, 3, and 4 in Supplement 1. The adjusted donation and transplant rates were comparable with the CALC rates used by CMS with median absolute differences less than 1%; this resulted in slight shifts of the threshold values (eTable 5 in Supplement 1).

**Figure 4.**
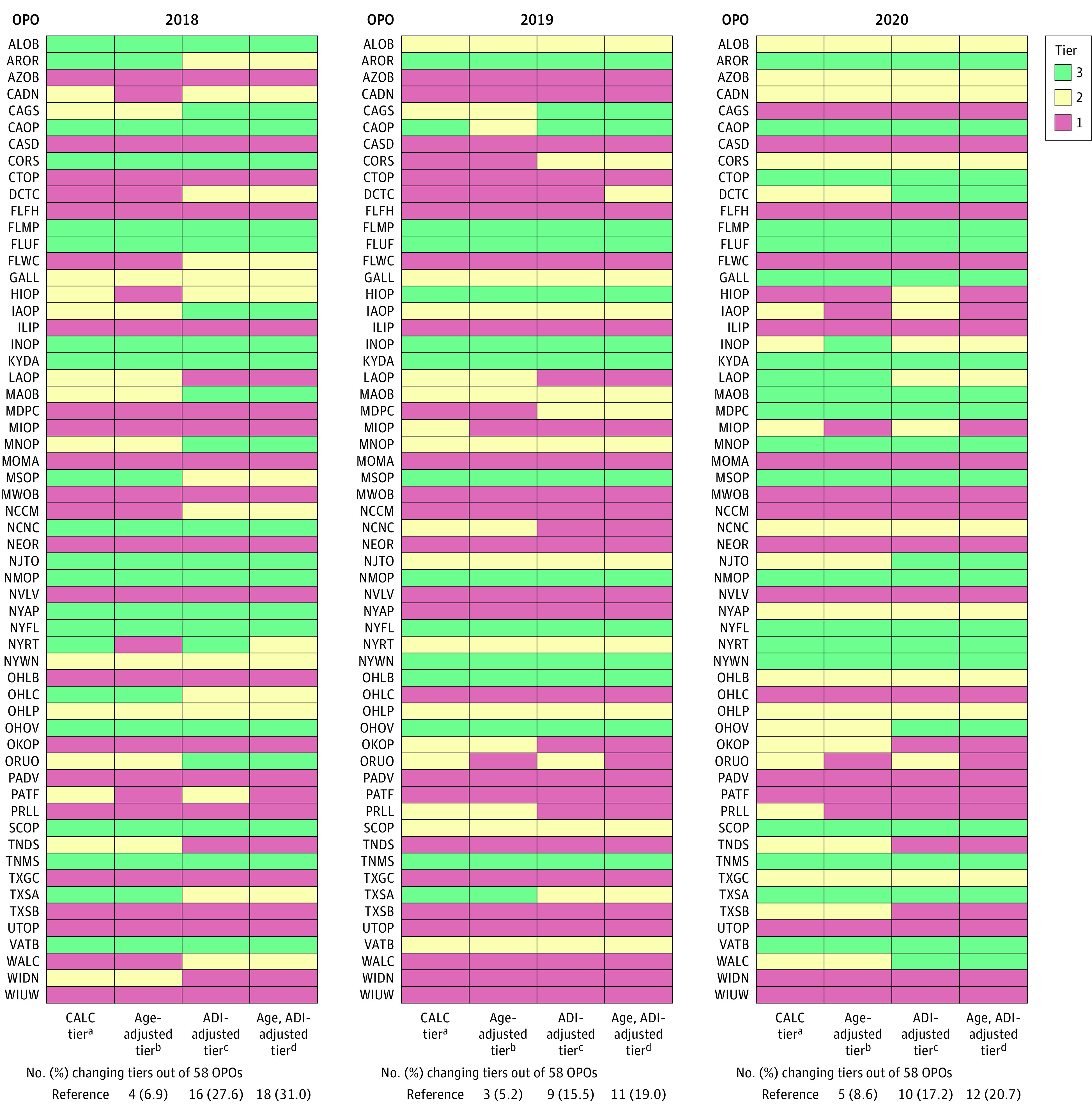
Age and Area Deprivation Index (ADI) Adjustment and Tier Assignments, 2018-2020 CALC indicates cause, age, and location consistent; OPO, Organ Procurement Organization. OPO codes and names are listed in eTable 6 in Supplement 1. ^a^Uses raw donation rate and age-adjusted transplant rate (or raw kidney transplant rate for HIOP [Legacy of Life Hawaii]); this is the tier used by CMS. ^b^Age-adjusted tier uses age-adjusted donation rate and age-adjusted transplant rate (or age-adjusted kidney transplant rate for HIOP). ^c^ADI-adjusted tier uses ADI-adjusted donation rate and age and ADI–adjusted transplant rate (or ADI-adjusted kidney transplant rate for HIOP). ^d^Age and ADI–adjusted tier uses age and ADI–adjusted donation rate and age and ADI–adjusted transplant rate (or age and ADI–adjusted kidney transplant rate for HIOP).

Compared with the CALC tier, additional age adjustment of donor and kidney transplant rates had reclassification rates of 6.9% (4 of 58) in 2018, 5.2% (3 of 58) in 2019, and 8.6% (5 of 58) in 2020, with 17.2% of OPOs (10 of 58) changing tiers at least once during the 3 years (Figure 4; OPO codes and names listed in eTable 6 in Supplement 1). Reclassification rates for ADI-adjusted tiers were 27.6% (16 of 58) in 2018, 15.5% (9 of 58) in 2019, and 17.2% (10 of 58) in 2020, with 44.8% of OPOs (26 of 58) changing tiers at least 1 of the 3 years. Age and ADI adjustment had reclassification rates of 31.0% (18 of 58) in 2018, 19.0% (11 of 58) in 2019, and 20.7% (12 of 58) in 2020, with 46.6% of OPOs (27 of 58) changing tiers at least once during the 3-year period.

Age adjustment alone resulted in 3 to 4 OPOs (5.2%-6.9%) moving into a lower tier than the CALC tier with most moving into tier 1; only 1 OPO moved into a higher tier (Table). ADI adjustment or age and ADI adjustment led to 5 to 9 OPOs (8.6%-15.5%) moving into a lower tier as compared with CALC tier, whereas 3 to 9 OPOs (5.2%-15.5%) moved into a higher tier. There were 3 to 7 OPOs (5.2%-12.1%) that moved from a CALC tier 2 or 3 to an adjusted tier 1, while up to 4 OPOs (6.9%) moved out of tier 1; 1 to 5 OPOs (1.7%-8.6%) moved out of tier 3 and up to 5 (8.6%) moved into tier 3 (Table). These changes were between tiers 1 and 2 with no OPO moving from a CALC tier 1 to an ADI-adjusted or an age and ADI–adjusted tier 3 or from CALC tier 3 to an adjusted tier 1 in any of the 3 years. Significant ranking reassignments are seen for both donor and transplant rates when assessed separately (eTable 7 in Supplement 1). Age-adjusted donation ranking is different than the CALC donation ranking at least once in the 3 years for 50% of OPOs (29 of 58). ADI-adjusted donation and transplantation rankings as well as age and ADI–adjusted donation and transplant rankings are different than the respective CALC ranking at least once for 46.6% of OPOs (27 of 58).

**Table.** Changes in Adjusted Tiers as Compared With CALC Tiers

Change as compared with CALC tier	OPOs, No. (%) (N=58)		
	2018	2019	2020
**Age-adjusted tier** ^a^			
Type of change			
No change	54 (93.1)	55 (94.8)	53 (91.4)
Moving into lower tier	4 (6.9)	3 (5.2)	4 (6.9)
Moving into higher tier	0	0	1 (1.7)
Moving into tier 1^b^	4 (6.9)	2 (3.4)	4 (6.9)
Moving out of tier 1^c^	0	0	0
Moving into tier 3^d^	0	0	1 (1.7)
Moving out of tier 3^e^	1 (1.7)^f^	1 (1.7)	0
**ADI-adjusted tier** ^g^			
Type of change			
No change	42 (72.4)	49 (84.5)	48 (82.8)
Moving into lower tier	7 (12.1)	6 (10.3)	5 (8.6)
Moving into higher tier	9 (15.5)	3 (5.2)	5 (8.6)
Moving into tier 1^b^	3 (5.2)	5 (8.6)	4 (6.9)
Moving out of tier 1^c^	4 (6.9)	2 (3.4)	1 (1.7)
Moving out of tier 3^e^	4 (6.9)	1 (1.7)	1 (1.7)
Moving into tier 3^d^	5 (8.6)	1 (1.7)	4 (6.9)
**Age and ADI–adjusted tier** ^h^			
Type of change			
No change	40 (69.0)	47 (81.0)	46 (79.3)
Moving into lower tier	9 (15.5)	7 (12.1)	8 (13.8)
Moving into higher tier	9 (15.5)	4 (6.9)	4 (6.9)
Moving into tier 1^b^	4 (6.9)	6 (10.3)	7 (12.1)
Moving out of tier 1^c^	4 (6.9)	3 (5.2)	0
Moving out of tier 3^e^	5 (8.6)	1 (1.7)	1 (1.7)
Moving into tier 3^d^	5 (8.6)	1 (1.7)	4 (6.9)

Abbreviations: ADI, area deprivation index; CALC, cause, age, and location consistent; OPO, Organ Procurement Organization.

^a^ Uses age-adjusted donation rate and age-adjusted transplant rate (or age-adjusted kidney transplant rate for HIOP [Life of Legacy Hawaii]).

^b^ CALC tier is 2 or 3 and adjusted tier is 1.

^c^ CALC tier is 1 and adjusted tier is 2 or 3.

^d^ CALC tier is 1 or 2 and adjusted tier is 3.

^e^ CALC tier is 3 and adjusted tier is 1 or 2.

^f^ This OPO moved out of tier 3 into tier 1, so it is included in the 4 OPOs seen in the row labeled, “Moving into tier 1.”

^g^ Uses ADI-adjusted donation rate and age and ADI–adjusted transplant rate (or ADI-adjusted kidney transplant rate for HIOP).

^h^ Uses age and ADI–adjusted donation rate and age and ADI–adjusted transplant rate (or age and ADI-adjusted kidney transplant rate for HIOP).

## Discussion

This study presents several prominent results addressing population characteristics that are associated with OPO performance measures. Findings illustrated that, using equivalent methods and data sources as CMS, adjustment for population characteristics significantly changed measured OPO performance. The primary shift in tier assignment was accounted by the ADI of applicable communities with a modest shift associated with decedents’ age. The measured OPO performance exhibited substantial changes, such that adjustment resulted in 15.5% to 27.6% tier reclassification based on ADI alone and 19.0% to 31.0% based on age and ADI on an annual basis. Cumulatively, 46.6% of OPOs changed tier at least once during the 3-year period with these adjustments. Findings also illustrated a significant correlation of obesity and diabetes burden with area deprivation. These findings suggest that differences in population characteristics can affect measured performance of OPOs and may represent substantial confounding factors that are not indicative of quality but rather influence donation and transplant rates. These results have important policy implications regarding the pending CMS guidelines for certifying and decertifying OPOs based on these quality measures.

Efforts to improve the donation and transplantation process are crucial for increasing transplant rates. The transplant system has well-documented inefficiencies and inequities that likely require intervention and policy implementation.^37^ Moreover, substantial evidence exists of regional and OPO variations in practice and important outcomes in donation and procurement processes.^38,39,40^ Thus, further efforts to improve quality and identify best practices of OPOs are important. However, measuring the quality of OPO performance accurately is challenging due to wide heterogeneity in geography, environment, and population characteristics. The current metrics assume that unmeasured factors affecting outcomes are equally distributed between DSAs. However, our study found substantial differences in population characteristics among DSAs, which significantly influenced the proposed metrics. These results suggest that current metrics are subject to confounding and/or that the differentiation of quality tiers as currently designed are highly sensitive to underlying factors.

Conditions of Coverage based on the current CMS Final Rule have substantial influence on the viability of OPOs and the transplantation system. Based on the stated policy, OPOs that are measured in lower tiers (2-3) may lose certification to perform services. Understanding potential underlying biases that may affect accurate quality measurement is critically important, considering the potential consequences for a substantial proportion of OPOs. Even with the expected imprecision of quality metrics that are designed to measure complex systems, the appropriate interpretation of results of these metrics should account for levels of uncertainty. However, with current policy, these metrics will lead to termination of OPOs, disruption of allocation systems, and potentially affect transplant opportunities for patients with end-stage organ disease.

The current study examined the outcome of including age of decedents, which is not included in the CMS donation rate measure, and ADI. Incorporating age into risk adjustment models used to measure performance had a modest influence on OPO measured performance with approximately 5% of OPOs shifting tiers. Evidence exists that age of decedents affects likelihood of organ donation and the willingness of transplants centers to accept organs for transplantation; moreover, the age of eligible donors varies across DSAs at a sufficient magnitude to alter performance measures.^22,41,42^ Given the objective nature of age and the evidence that age influences donation rate, it seems straightforward that these could be accounted for in quality performance measures of OPOs. However, the factor that altered the overall risk-adjusted OPO tier assignment more profoundly was ADI.

The mechanisms for changes in measured OPO performance with ADI adjustment are likely multifactorial. As displayed in the findings, comorbidity burden is significantly associated with ADI and might represent underlying risk factors that affect donation or transplantation, and which are considered in clinical care but not accounted for in current metrics. ADI and similar indices have been shown in numerous studies to affect processes of care and patient outcomes^4,43,44,45,46^ Of note, ADI does not include race and ethnicity in the calculation, and while there is an association of minoritized race and ethnicity and residence in higher deprivation community, race and ethnicity are not factors in the current analysis. Thus, while ADI may represent multiple sources of risk related to disease burden, health care resources and access to health care, it is a robust indicator of variations in health and socioeconomic status. Importantly for this study, ADI may represent factors directly associated with donation and transplantation rates that are not attributable to OPO performance. This study also highlights a need to further understand the drivers of the lower likelihood of donation and transplantation in higher deprivation communities and to develop meaningful interventions.

The National Quality Forum (NQF), whose endorsement is often a prerequisite for CMS implementation, addressed the appropriateness of adjusting for sociodemographic risk factors in quality metrics. The NQF recommended inclusion of sociodemographic factors in risk adjustment of performance measures when a conceptual framework exists and suggests stratification of these factors to improve understanding of variations in practice.^47^ As such, adjustment for ADI or ADI and age is consistent with the NQF-endorsed recommendation.

### Limitations

There are limitations of this study that should be considered for interpretation. Although we used the same data sources and methods as CMS, there were modest discrepancies in our calculations as compared with CMS. These differences were minor and may be affected by counties given waivers for inclusion. However, as we used these data consistently throughout the analysis, differences would not account for changes in measured performance and tier assignment in our analysis. We selected to use ADI as an indicator for variations in underlying population characteristics. Yet, there are other indices that express similar domains of health burden and socioeconomic status that may be equally useful or potentially modify current findings. While many of these indices (eg, CDC Social Vulnerability Index or Economic Innovation Group’s Distressed Community Index) rely on similar census data, they have different algorithms and it is plausible that using them would modify findings.^48,49^ Also, other forms of risk adjustment such as for population-based comorbidities may be considered instead. Furthermore, some of the observed findings may be based on changes in performance in certain regions such as acceptance behaviors at transplant centers as well OPO-specific practices which would directly affect transplantation rates.^50,51^ Additionally, policy by CMS was evolving over the study period and performance may have fluctuated because of pending rules.

## Conclusions

In this cross-sectional study of population characteristics and OPO performance metrics, we found that adjusting for area deprivation and age significantly changed OPO measured performance and tier classifications. The possible conclusions are that (1) OPOs that serve highly distressed communities were systematically lower performers or (2) population characteristics represent substantial underlying factors that influenced measured quality but may not have been indicative of performance. Findings also indicated that based on current Conditions of Coverage, OPOs that served communities with higher prevalence of comorbid burden and deprivation were more likely to be evaluated with lower performance and lose certification in future years, which may have additional unintended downstream consequences for these communities. The results of this study hold importance for potential policy modifications and warrant further evaluation of OPO quality metrics.

## Notes

Supplement 1. **eMethods.** Methodology for ADI- and Age, ADI-Adjustment of Donation and Transplantation Rates **eTable 1.** Donation Rates, Organ Transplant Rates and Tier Assignments for 57 OPOs, 2018 **eTable 2.** Donation Rates, Organ Transplant Rates and Tier Assignments for 57 OPOs, 2019 **eTable 3.** Donation Rates, Organ Transplant Rates and Tier Assignments for 57 OPOs, 2020 **eTable 4.** Donation Rates, Kidney Transplant Rates and Tier Assignments for HIOP, 2018-2020 **eTable 5.** Threshold Values Used for Tier Assignments **eTable 6.** List of Organ Procurement Organization (OPO) Codes and Names **eTable 7.** Reclassification Rates as Compared to CALC Rankings for Donation and Transplant Rates

Supplement 2.
Data Sharing Statement
