# Error in End Matter

**DOI:** 10.1001/jamanetworkopen.2023.46625

**Published:** 2023-11-13

In the Original Investigation titled “Population Characteristics and Organ Procurement Organization Performance Metrics,”^1^ published October 3, 2023, the end matter’s Additional Contributions section was missing information about J. Thomas Rosenthal, MD. This article has been corrected.^1^
