# Supplementary material for: Population Characteristics and Organ Procurement Organization Performance Metrics
Source: JAMA Netw Open. 2023 Oct 3;6(10):e2336749. doi: 10.1001/jamanetworkopen.2023.36749 (PMC10548299; doi:10.1001/jamanetworkopen.2023.36749)
Supplement: Supplement 1. — eMethods. Methodology for ADI- and Age, ADI-Adjustment of Donation and Transplantation Rates eTable 1. Donation Rates, Organ Transplant Rates and Tier Assignments for 57 OPOs, 2018 eTable 2. Donation Rates, Organ Transplant Rates and Tier Assignments for 57 OPOs, 2019 eTable 3. Donation Rates, Organ Transplant Rates and Tier Assignments for 57 OPOs, 2020 eTable 4. Donation Rates, Kidney Transplant Rates and Tier Assignments for HIOP, 2018-2020 eTable 5. Threshold Values Used for Tier Assignments eTable 6. List of Organ Procurement Organization (OPO) Codes and Names eTable 7. Reclassification Rates as Compared to CALC Rankings for Donation and Transplant Rates [file jamanetwopen-e2336749-s001.pdf]

Supplemental Online Content

Lopez R, Mohan S, Schold JD. Population characteristics and organ procurement organization performance metrics. *JAMA Netw Open.* 2023;6(10):e2336749. doi:10.1001/jamanetworkopen.2023.36749

eMethods. Methodology for ADI- and age, ADI-adjustment of donation and transplantation rates

eTable 1. Donation Rates, Organ Transplant Rates and Tier Assignments for 57 OPOs, 2018

eTable 2. Donation Rates, Organ Transplant Rates and Tier Assignments for 57 OPOs, 2019

eTable 3. Donation Rates, Organ Transplant Rates and Tier Assignments for 57 OPOs, 2020

eTable 4. Donation Rates, Kidney Transplant Rates and Tier Assignments for HIOP, 2018-2020

eTable 5. Threshold Values Used for Tier Assignments

eTable 6. List of Organ Procurement Organization (OPO) Codes and Names

eTable 7. Reclassification Rates as Compared to CALC Rankings for Donation and Transplant Rates

This supplemental material has been provided by the authors to give readers additional information about their work.

eMethods. Methodology for ADI- and age, ADI-adjustment of donation and transplantation rates

Following the age-adjustment approach used by CMS, we used indirect standardization for ADI-adjustment of donation and transplant rates. The steps are as follows:

1. ADI quintiles (1-5) are used for the adjusted rate calculation. As explained above, potential donors, donors and transplanted organs are categorized based on the ADI corresponding to the place of residence.
2. Calculate national ADI-specific donation and transplant rates for each ADI quintile.
3. For each OPO, calculate the expected donation and transplant rates as $\sum_{g=1}^{G} d_g \, R_g / \sum_g d_g$. Where d_g is the number of potential donors in the OPO in ADI group g, R_g is the ADI-specific national donation or transplant rate in ADI group g, and $\sum_g d_g$ is the OPO's total number of potential donors.
4. For each OPO, calculate the OPO ADI-adjusted donation and transplant rates as $\left({}^{OPO\ Observed\ Rate}/_{OPO\ Expected\ Rate} \right) \times National\ Transplant\ Rate$

We also followed the above methodology to adjust the donation and transplant rates for both age and ADI. This adjustment was done using the 15 age categories (<1, 1-5, 6-11, 12-17, 18-24, 25-29, 30-34, 35-39, 40-44, 45-49, 50-54, 55-59, 60-64, 65-69, 70-75) used in the new rule and the ADI quintiles for a total of 75 age-ADI categories.

© 2023 Lopez R et al. *JAMA Network Open.*

eTable 1. Donation rates, organ transplant rates and tier assignments for 57 OPOs[a], 2018

	Donation rates								Transplant rates				Tiers			
	CALC (unadjusted)		Age-adjusted		ADI-adjusted		Age, ADI-adjusted		CALC (age-adjusted)		Age, ADI-adjusted					
OPO	Rate	95% CI[b]	Rate	95% CI[b]	Rate	95% CI[b]	Rate	95% CI[b]	Rate	95% CI[b]	Rate	95% CI[b]	CALC tier[c]	Age-adjusted tier[d]	ADI-adjusted tier[e]	Age, ADI-adjusted tier[f]
CTOP	17.99	21.41	16.55	19.69	16.40	19.51	14.85	17.67	49.11	54.53	43.72	48.55	1	1	1	1
MWOB	17.85	19.62	16.90	18.64	17.93	19.78	16.94	18.69	52.95	55.99	53.04	56.08	1	1	1	1
UTOP	15.43	17.79	13.88	16	14.41	16.61	12.84	14.81	46.57	50.37	42.89	46.39	1	1	1	1
PADV	16.45	17.54	15.22	16.28	16.14	17.26	15.07	16.13	45.10	46.91	44.92	46.72	1	1	1	1
NEOR	14.16	17.17	17.30	20.83	13.29	16.01	16.04	19.31	58.76	65.39	53.83	59.89	1	1	1	1
WIUW	13.79	15.75	14.16	16.16	13.26	15.13	13.68	15.61	48.68	52.34	46.92	50.44	1	1	1	1
CASD	13.42	15.39	15.13	17.33	12.38	14.18	13.74	15.74	48.97	52.97	44.14	47.75	1	1	1	1
ILIP	13.19	14.22	13.67	14.76	12.62	13.62	13.11	14.16	42.35	44.28	40.63	42.48	1	1	1	1
FLFH	12.42	13.97	11.93	13.41	11.73	13.19	11.26	12.66	39.90	42.59	37.66	40.2	1	1	1	1
NVLV	12.03	13.87	12.44	14.29	12.64	14.52	12.99	14.92	45.24	48.8	47.81	51.58	1	1	1	1
NCCM	11.88	13.79	10.77	12.46	11.74	13.58	10.56	12.21	34.29	37.28	33.44	36.36	1	1	2	2
AZOB	12.41	13.68	10.95	12.08	12.17	13.43	10.77	11.89	38.65	40.73	38.03	40.08	1	1	1	1
MDPC	11.89	13.55	10.42	11.85	11.74	13.35	10.31	11.72	36.33	38.96	35.71	38.29	1	1	1	1
TXGC	11.96	12.96	11.84	12.85	12.16	13.2	12.04	13.06	39.61	41.46	40.51	42.4	1	1	1	1
DCTC	11.10	12.73	9.96	11.39	10.14	11.59	8.99	10.27	35.92	38.58	32.16	34.54	1	1	2	2
MOMA	11.27	12.64	10.45	11.71	11.71	13.11	10.85	12.16	34.63	36.9	35.81	38.15	1	1	1	1
OKOP	11.15	12.56	12.26	13.79	11.86	13.34	13.11	14.74	38.94	41.7	41.94	44.91	1	1	1	1
TXSB	11.13	12.11	10.94	11.91	11.72	12.76	11.53	12.56	35.42	37.16	37.60	39.45	1	1	1	1
OHLB	10.63	12.07	10.73	12.15	10.88	12.33	11.07	12.54	35.05	37.62	36.35	39.01	1	1	1	1
WALC	10.70	11.8	10.77	11.87	9.99	11.01	9.98	11.01	35.47	37.45	32.76	34.59	1	1	2	2
PRLL	9.95	11.71	13.35	15.6	12.16	14.21	16.13	18.85	40.65	44.66	51.01	56.04	1	1	1	1
FLWC	10.50	11.63	11.16	12.36	10.07	11.14	10.80	11.96	36.56	38.74	35.55	37.67	1	1	2	2
MIOP	10.50	11.47	11.37	12.42	10.94	11.95	11.99	13.1	39.14	41.09	41.16	43.22	1	1	1	1
WIDN	10.56	12.66	10.20	12.11	11.06	13.14	11.01	13.08	32.39	35.82	34.85	38.54	2	2	1	1
NYWN	9.75	12.32	10.79	13.38	9.48	11.75	10.54	13.07	30.50	34.83	29.64	33.85	2	2	2	2
LAOP	10.72	12.04	10.18	11.41	12.02	13.48	11.42	12.8	33.13	35.35	37.29	39.79	2	2	1	1
TNDS	10.25	11.27	10.34	11.36	10.87	11.94	10.91	11.98	34.53	36.4	36.45	38.42	2	2	1	1
CADN	10.17	11.12	10.60	11.59	9.88	10.8	10.20	11.15	36.81	38.67	35.25	37.02	2	1	2	2
MAOB	10.12	11.08	10.31	11.28	9.37	10.26	9.46	10.36	32.31	34.05	29.54	31.13	2	2	3	3
GALL	10.07	11.06	9.92	10.89	10.50	11.53	10.38	11.39	31.85	33.6	33.40	35.24	2	2	2	2

© 2023 Lopez R et al. *JAMA Network Open.*

eTable 1. Donation rates, organ transplant rates and tier assignments for 57 OPOs[a], 2018

	Donation rates								Transplant rates				Tiers			
	CALC (unadjusted)		Age-adjusted		ADI-adjusted		Age, ADI-adjusted		CALC (age-adjusted)		Age, ADI-adjusted					
**OPO**	**Rate**	**95% CI[b]**	**Rate**	**95% CI[b]**	**Rate**	**95% CI[b]**	**Rate**	**95% CI[b]**	**Rate**	**95% CI[b]**	**Rate**	**95% CI[b]**	**CALC tier[c]**	**Age-adjusted tier[d]**	**ADI-adjusted tier[e]**	**Age, ADI-adjusted tier[f]**
OHLP	9.49	10.95	9.28	10.65	9.57	10.98	9.34	10.72	30.73	33.22	30.92	33.44	2	2	2	2
MNOP	9.50	10.73	9.40	10.58	8.86	9.97	8.76	9.86	30.56	32.66	28.45	30.41	2	2	3	3
PATF	9.43	10.57	10.80	12.07	9.65	10.78	11.12	12.43	38.06	40.46	39.19	41.67	2	1	2	1
ORUO	8.61	9.93	9.67	11.09	8.18	9.38	9.12	10.46	30.82	33.36	29.01	31.4	2	2	3	3
CAGS	8.07	9.79	8.94	10.7	7.50	8.97	8.14	9.74	30.99	34.3	28.04	31.03	2	2	3	3
IAOP	7.94	9.62	8.45	10.09	7.64	9.13	8.01	9.58	29.37	32.44	27.73	30.63	2	2	3	3
CORS	12.03	13.65	9.69	10.98	11.21	12.7	8.91	10.09	29.60	31.8	27.06	29.07	3	3	3	3
OHOV	10.17	12.27	8.48	10.12	9.62	11.48	8.01	9.57	26.75	29.64	25.16	27.87	3	3	3	3
OHLC	9.30	11.26	8.61	10.3	9.68	11.58	9.03	10.81	27.63	30.61	29.01	32.13	3	3	2	2
SCOP	9.78	11.04	8.96	10.08	10.14	11.41	9.24	10.4	28.26	30.24	29.35	31.4	3	3	3	3
NMOP	8.04	10.23	7.92	9.86	8.78	10.94	8.77	10.92	23.76	27.11	26.63	30.38	3	3	3	3
FLUF	8.74	9.94	8.48	9.6	8.91	10.1	8.66	9.81	26.84	28.85	27.39	29.44	3	3	3	3
MSOP	8.29	9.86	8.90	10.47	9.70	11.42	10.58	12.46	28.34	31.22	34.01	37.46	3	3	2	2
TXSA	8.68	9.82	8.42	9.5	9.14	10.31	8.79	9.92	28.87	30.85	30.33	32.42	3	3	2	2
NYRT	8.66	9.5	10.83	11.88	8.40	9.2	10.49	11.5	36.63	38.62	35.42	37.34	3	1	3	2
VATB	7.95	9.04	8.26	9.35	7.98	9.03	8.27	9.36	27.87	29.88	27.90	29.92	3	3	3	3
NYAP	7.21	9.04	8.96	11	6.93	8.51	8.48	10.42	25.47	28.97	23.98	27.28	3	3	3	3
NJTO	7.88	8.96	8.20	9.27	7.27	8.22	7.52	8.51	28.67	30.71	26.25	28.11	3	3	3	3
NYFL	7.21	8.94	7.53	9.17	7.02	8.56	7.42	9.03	25.01	27.99	24.62	27.55	3	3	3	3
CAOP	8.29	8.92	8.79	9.46	7.69	8.28	8.06	8.68	31.03	32.3	28.26	29.42	3	3	3	3
INOP	7.79	8.81	6.89	7.76	8.36	9.42	7.47	8.42	25.23	26.88	27.36	29.15	3	3	3	3
AROR	7.06	8.69	7.49	9.06	7.88	9.53	8.31	10.06	26.08	29.05	29.07	32.39	3	3	2	2
NCNC	7.58	8.52	7.95	8.9	8.19	9.16	8.60	9.63	26.93	28.69	29.30	31.21	3	3	3	3
KYDA	7.15	8.25	6.91	7.91	7.63	8.73	7.33	8.39	24.41	26.3	26.02	28.03	3	3	3	3
TNMS	6.66	8.19	6.12	7.39	7.81	9.44	7.16	8.66	19.14	21.39	22.30	24.92	3	3	3	3
FLMP	6.87	7.86	7.10	8.07	6.69	7.6	6.90	7.84	24.07	25.85	23.47	25.21	3	3	3	3
ALOB	5.61	6.4	4.45	5.04	6.20	7.02	4.95	5.61	13.06	14.05	14.55	15.65	3	3	3	3

ADI: area deprivation index; CALC: cause, age, location, consistent; CI: confidence interval; OPO: organ procurement organization

[a] Excludes HIOP which can be seen on Supplemental Table 4
[b] 95% upper confidence limit
[c] Uses unadjusted donation rate and age-adjusted transplant rate.
[d] Uses age-adjusted donation rate and age-adjusted transplant rate.
[d] Uses ADI-adjusted donation rate and age, ADI-adjusted transplant rate.
[f] Uses Age, ADI-adjusted donation rate and age, ADI-adjusted transplant rate.

© 2023 Lopez R et al. *JAMA Network Open.*

eTable 2. Donation rates, organ transplant rates and tier assignments for 57 OPOs[a], 2019

OPO	Donation rates								Transplant rates				Tiers			
	CALC (unadjusted)		Age-adjusted		ADI-adjusted		Age, ADI-adjusted		CALC (age-adjusted)		Age, ADI-adjusted		CALC tier[c]	Age-adjusted tier[d]	ADI-adjusted tier[e]	Age, ADI-adjusted tier[f]
	Rate	95% CI[b]	Rate	95% CI[b]	Rate	95% CI[b]	Rate	95% CI[b]	Rate	95% CI[b]	Rate	95% CI[b]				
CTOP	19.01	22.77	18.42	22.06	17.80	21.33	17.12	20.51	60.20	66.72	55.81	61.85	1	1	1	1
MWOB	17.76	19.47	16.46	18.11	17.80	19.59	16.56	18.23	56.36	59.4	56.51	59.55	1	1	1	1
PADV	17.92	19.06	17.15	18.32	17.80	19.01	17.16	18.32	54.06	56.12	54.27	56.33	1	1	1	1
NVLV	15.93	18.01	16.00	18.11	16.48	18.64	16.69	18.88	51.09	54.91	53.25	57.23	1	1	1	1
UTOP	15.59	17.97	12.66	14.59	14.85	17.11	11.98	13.81	40.55	43.93	38.26	41.45	1	1	1	1
MOMA	15.86	17.45	15.59	17.19	16.27	17.94	16.01	17.66	46.71	49.46	48.12	50.96	1	1	1	1
WIDN	13.99	16.3	14.06	16.34	14.12	16.4	14.24	16.55	44.73	48.81	45.40	49.54	1	1	1	1
NEOR	13.18	16	14.13	17.01	12.64	15.21	13.59	16.37	48.62	53.98	46.72	51.86	1	1	1	1
CORS	13.94	15.62	11.86	13.3	13.22	14.83	11.17	12.52	37.08	39.58	34.86	37.21	1	1	2	2
NCCM	13.35	15.46	12.07	13.95	13.18	15.23	11.81	13.65	43.12	46.61	42.18	45.6	1	1	1	1
CASD	13.37	15.38	13.50	15.5	12.64	14.52	12.55	14.41	47.29	51.04	43.79	47.26	1	1	1	1
FLFH	13.41	15.02	12.26	13.74	12.78	14.32	11.79	13.21	40.53	43.16	38.87	41.41	1	1	1	1
MDPC	13.14	14.9	11.72	13.28	12.92	14.64	11.48	13.02	36.09	38.81	35.22	37.87	1	1	2	2
WIUW	12.97	14.83	14.83	16.94	12.56	14.34	14.46	16.51	50.98	54.9	49.56	53.37	1	1	1	1
AZOB	13.51	14.8	11.07	12.14	13.23	14.51	11.05	12.12	37.21	39.12	37.05	38.95	1	1	1	1
ILIP	13.18	14.21	13.95	15.06	12.67	13.69	13.58	14.66	44.49	46.5	43.14	45.09	1	1	1	1
OHLC	11.95	14.07	10.82	12.68	12.16	14.25	11.07	12.97	35.86	39.2	36.57	39.98	1	1	1	1
TXSB	12.76	13.79	11.87	12.84	13.28	14.37	12.41	13.43	38.17	39.9	40.13	41.95	1	1	1	1
WALC	12.33	13.51	12.77	13.99	11.79	12.92	12.14	13.31	40.35	42.53	38.25	40.31	1	1	1	1
CADN	12.41	13.43	13.19	14.29	12.12	13.13	12.77	13.83	42.47	44.47	40.93	42.85	1	1	1	1
NYAP	10.87	13.14	12.73	15.24	10.48	12.54	12.26	14.67	38.77	43.17	37.10	41.31	1	1	1	1
TXGC	11.93	12.89	11.22	12.13	12.10	13.09	11.38	12.31	37.73	39.4	38.29	39.98	1	1	1	1
FLWC	11.61	12.81	12.79	14.11	11.16	12.31	12.47	13.76	41.17	43.57	40.10	42.43	1	1	1	1
DCTC	11.13	12.74	10.68	12.19	10.44	11.91	9.90	11.29	39.24	42.13	36.17	38.82	1	1	1	2
TNDS	11.36	12.4	11.27	12.3	11.77	12.85	11.65	12.72	37.47	39.36	38.81	40.76	1	1	1	1
PATF	11.19	12.39	12.72	14.09	11.37	12.59	12.93	14.32	41.30	43.8	41.98	44.52	1	1	1	1
LAOP	12.96	14.37	11.01	12.21	14.09	15.63	11.86	13.16	36.36	38.53	39.47	41.83	2	2	1	1
GALL	11.70	12.74	11.10	12.09	12.24	13.34	11.51	12.55	34.55	36.3	36.01	37.84	2	2	2	2
OKOP	11.13	12.56	11.34	12.78	11.62	13.09	11.88	13.38	34.73	37.25	36.43	39.08	2	2	1	1
MNOP	11.07	12.38	11.03	12.32	10.53	11.76	10.50	11.73	35.28	37.58	33.47	35.65	2	2	2	2
OHLP	10.70	12.25	10.25	11.69	10.66	12.16	10.30	11.75	31.90	34.44	32.04	34.6	2	2	2	2

© 2023 Lopez R et al. *JAMA Network Open.*

eTable 2. Donation rates, organ transplant rates and tier assignments for 57 OPOs[a], 2019

OPO	Donation rates								Transplant rates				Tiers			
	CALC (unadjusted)		Age-adjusted		ADI-adjusted		Age, ADI-adjusted		CALC (age-adjusted)		Age, ADI-adjusted					
	Rate	95% CI[b]	Rate	95% CI[b]	Rate	95% CI[b]	Rate	95% CI[b]	Rate	95% CI[b]	Rate	95% CI[b]	CALC tier[c]	Age-adjusted tier[d]	ADI-adjusted tier[e]	Age, ADI-adjusted tier[f]
SCOP	10.56	11.9	9.30	10.45	10.95	12.32	9.68	10.88	31.68	33.79	33.11	35.32	2	2	2	2
NCNC	10.66	11.77	10.94	12.07	11.27	12.44	11.61	12.82	35.89	37.95	38.26	40.46	2	2	1	1
MIOP	10.64	11.62	11.66	12.74	10.79	11.79	11.91	13.01	39.30	41.29	39.95	41.98	2	1	1	1
IAOP	9.77	11.58	10.09	11.85	9.43	11.09	9.68	11.38	33.27	36.48	31.83	34.9	2	2	2	2
ALOB	10.16	11.41	10.30	11.54	10.91	12.22	11.01	12.34	32.94	35.17	35.33	37.73	2	2	2	2
MAOB	10.38	11.33	10.60	11.58	9.81	10.71	9.91	10.82	33.99	35.74	31.61	33.24	2	2	2	2
PRLL	9.52	11.27	12.51	14.68	11.93	14	15.45	18.13	31.55	35.08	41.27	45.88	2	2	1	1
NJTO	9.96	11.18	10.61	11.88	9.35	10.47	9.96	11.15	34.04	36.33	31.73	33.86	2	2	2	2
ORUO	9.66	11.08	11.48	13.1	9.25	10.56	10.90	12.45	38.75	41.8	36.60	39.48	2	1	2	1
CAGS	8.64	10.43	8.79	10.48	8.19	9.77	8.24	9.83	29.29	32.39	27.27	30.16	2	2	3	3
VATB	9.08	10.24	9.86	11.08	9.09	10.22	9.82	11.04	34.67	37	34.48	36.79	2	2	2	2
NYRT	9.24	10.1	10.79	11.78	9.13	9.97	10.69	11.68	33.76	35.55	33.51	35.28	2	2	2	2
OHOV	10.91	13.12	8.96	10.67	10.46	12.46	8.70	10.36	29.00	32.04	28.15	31.1	3	3	3	3
NMOP	9.51	11.78	8.62	10.51	10.28	12.54	9.40	11.47	24.35	27.5	26.77	30.23	3	3	3	3
FLUF	10.42	11.75	9.48	10.66	10.48	11.78	9.56	10.75	28.76	30.79	29.00	31.05	3	3	3	3
OHLB	9.48	10.87	8.96	10.22	9.58	10.93	9.13	10.42	29.34	31.61	29.86	32.17	3	3	3	3
TXSA	9.53	10.69	9.16	10.26	10.03	11.23	9.54	10.68	30.02	31.99	31.42	33.48	3	3	2	2
NYFL	8.22	10.14	8.69	10.55	8.04	9.76	8.63	10.48	28.28	31.64	27.99	31.33	3	3	3	3
INOP	8.96	10.07	7.88	8.83	9.30	10.42	8.21	9.19	27.90	29.67	29.01	30.85	3	3	3	3
CAOP	9.18	9.86	9.75	10.46	8.72	9.36	9.20	9.87	33.84	35.19	31.71	32.98	3	2	3	3
NYWN	7.11	9.54	8.14	10.54	6.91	8.96	8.10	10.49	21.16	25.08	21.09	25	3	3	3	3
MSOP	7.08	8.57	7.84	9.36	8.03	9.58	8.74	10.43	25.35	28.1	28.59	31.69	3	3	3	3
FLMP	7.42	8.47	7.74	8.79	7.25	8.23	7.62	8.65	27.10	29.06	26.57	28.49	3	3	3	3
KYDA	6.96	8.03	7.56	8.65	7.40	8.47	8.01	9.17	26.81	28.91	28.66	30.91	3	3	3	3
TNMS	6.43	7.97	6.41	7.79	7.18	8.73	7.11	8.64	20.60	23.1	23.09	25.89	3	3	3	3
AROR	6.42	7.87	7.18	8.66	6.95	8.37	7.67	9.24	24.41	27.17	26.23	29.2	3	3	3	3

ADI: area deprivation index; CALC: cause, age, location, consistent; CI: confidence interval; OPO: organ procurement organization

[a] Excludes HIOP which can be seen on Supplemental Table 4

[b] 95% upper confidence limit

[c] Uses unadjusted donation rate and age-adjusted transplant rate.

[d] Uses age-adjusted donation rate and age-adjusted transplant rate.

[d] Uses ADI-adjusted donation rate and age, ADI-adjusted transplant rate.

[f] Uses Age, ADI-adjusted donation rate and age, ADI-adjusted transplant rate.

© 2023 Lopez R et al. *JAMA Network Open.*

eTable 3. Donation rates, organ transplant rates and tier assignments for 57 OPOs[a], 2020

	Donation rates								Transplant rates				Tiers			
	CALC (unadjusted)		Age-adjusted		ADI-adjusted		Age, ADI-adjusted		CALC (age-adjusted)		Age, ADI-adjusted					
OPO	Rate	95% CI[b]	Rate	95% CI[b]	Rate	95% CI[b]	Rate	95% CI[b]	Rate	95% CI[b]	Rate	95% CI[b]	CALC tier[c]	Age-adjusted tier[d]	ADI-adjusted tier[e]	Age, ADI-adjusted tier[f]
NVLV	19.14	21.41	19.57	21.99	19.70	22.14	19.88	22.34	56.88	61.03	57.81	62.02	1	1	1	1
MWOB	18.97	20.66	18.82	20.59	18.93	20.71	18.82	20.6	54.08	57.05	54.02	56.98	1	1	1	1
UTOP	17.79	20.21	13.93	15.87	16.89	19.23	13.22	15.05	45.19	48.56	43.34	46.57	1	1	1	1
PADV	16.94	18.05	16.66	17.82	16.83	18.01	16.80	17.97	48.21	50.18	49.10	51.1	1	1	1	1
CASD	15.62	17.89	14.89	17.06	14.73	16.89	13.84	15.86	49.38	53.3	46.30	49.97	1	1	1	1
WIUW	15.81	17.78	16.01	18.03	15.32	17.26	15.55	17.51	53.05	56.72	51.38	54.93	1	1	1	1
MOMA	15.75	17.3	15.58	17.16	16.10	17.73	15.78	17.38	52.93	55.84	53.35	56.29	1	1	1	1
OHLC	14.55	16.87	13.90	16.09	14.80	17.13	14.13	16.35	43.81	47.66	44.07	47.94	1	1	1	1
NCCM	14.76	16.81	12.80	14.58	14.61	16.63	12.56	14.3	40.94	44.07	40.14	43.2	1	1	1	1
CAGS	14.63	16.73	13.86	15.84	13.80	15.78	13.00	14.86	41.35	44.76	38.99	42.21	1	1	1	1
NEOR	13.23	15.95	14.79	17.69	12.59	15.07	13.96	16.7	49.57	54.97	46.84	51.94	1	1	1	1
FLFH	13.80	15.38	12.83	14.31	13.15	14.67	12.42	13.86	43.62	46.34	42.36	44.99	1	1	1	1
WIDN	13.05	15.17	13.37	15.5	13.32	15.44	13.86	16.06	44.42	48.31	45.34	49.31	1	1	1	1
PATF	13.40	14.67	15.16	16.63	13.62	14.94	15.38	16.87	45.86	48.45	46.41	49.03	1	1	1	1
FLWC	12.65	13.87	14.10	15.47	12.20	13.38	13.86	15.2	46.76	49.28	46.04	48.52	1	1	1	1
ILIP	12.87	13.86	13.16	14.19	12.36	13.33	12.81	13.82	41.04	42.87	40.00	41.78	1	1	1	1
CORS	14.28	15.92	12.33	13.77	13.52	15.09	11.57	12.92	37.02	39.46	34.94	37.24	2	2	2	2
AZOB	12.78	14	11.28	12.37	12.56	13.78	11.35	12.45	36.78	38.72	36.94	38.88	2	2	2	2
OHLP	11.80	13.39	10.59	12	11.77	13.33	10.65	12.07	35.31	37.86	35.31	37.86	2	2	2	2
OKOP	11.85	13.3	12.58	14.1	12.32	13.8	12.98	14.55	36.64	39.25	37.78	40.47	2	2	1	1
OHOV	11.08	13.28	10.19	12.12	10.63	12.64	9.93	11.8	32.81	36.25	32.06	35.43	2	2	3	3
TNDS	12.07	13.11	12.00	13.05	12.54	13.64	12.49	13.58	37.99	39.86	39.25	41.18	2	2	1	1
IAOP	11.10	13.06	12.07	14.12	10.76	12.59	11.65	13.63	38.94	42.65	37.63	41.22	2	1	2	1
CADN	12.01	13.01	12.07	13.1	11.72	12.72	11.73	12.72	38.23	40.06	37.14	38.91	2	2	2	2
ORUO	11.51	13	12.23	13.8	11.05	12.46	11.70	13.19	39.80	42.63	38.04	40.75	2	1	2	1
TXSB	11.96	12.95	11.72	12.7	12.44	13.49	12.18	13.21	39.91	41.73	41.52	43.41	2	2	1	1
PRLL	10.85	12.68	15.76	18.32	13.44	15.61	19.19	22.31	38.39	42.52	49.10	54.38	2	1	1	1
NYAP	10.51	12.67	12.12	14.47	10.15	12.12	11.74	14.02	35.75	39.83	34.80	38.77	2	2	2	2
TXGC	11.62	12.55	11.17	12.07	11.75	12.7	11.26	12.17	37.01	38.65	37.32	38.98	2	2	2	2
WALC	11.39	12.5	11.54	12.67	10.89	11.95	10.98	12.05	34.78	36.72	33.29	35.14	2	2	3	3
NCNC	11.21	12.31	10.97	12.04	11.85	13.01	11.50	12.62	34.13	36.02	35.60	37.57	2	2	2	2
MIOP	11.10	12.09	12.49	13.61	11.32	12.33	12.84	13.99	39.51	41.52	39.99	42.03	2	1	2	1
NJTO	10.77	12.03	11.67	13.01	10.12	11.29	11.02	12.29	35.01	37.37	33.15	35.39	2	2	3	3

© 2023 Lopez R et al. *JAMA Network Open.*

eTable 3. Donation rates, organ transplant rates and tier assignments for 57 OPOs[a], 2020

OPO	Donation rates								Transplant rates				Tiers			
	CALC (unadjusted)		Age-adjusted		ADI-adjusted		Age, ADI-adjusted		CALC (age-adjusted)		Age, ADI-adjusted					
	Rate	95% CI[b]	Rate	95% CI[b]	Rate	95% CI[b]	Rate	95% CI[b]	Rate	95% CI[b]	Rate	95% CI[b]	CALC tier[c]	Age-adjusted tier[d]	ADI-adjusted tier[e]	Age, ADI-adjusted tier[f]
INOP	10.81	11.97	9.88	10.93	11.25	12.45	10.18	11.26	34.84	36.8	35.30	37.28	2	3	2	2
DCTC	9.95	11.5	9.81	11.28	9.31	10.71	9.07	10.43	33.76	36.5	31.30	33.84	2	2	3	3
ALOB	10.18	11.44	10.89	12.22	10.89	12.22	11.57	12.98	35.06	37.45	36.86	39.38	2	2	2	2
OHLB	10.05	11.41	9.87	11.18	10.13	11.47	9.99	11.3	33.63	36.03	33.95	36.37	2	2	2	2
MDPC	11.69	13.4	10.72	12.26	11.47	13.12	10.41	11.91	32.36	35	31.24	33.8	3	3	3	3
NMOP	10.61	12.95	9.11	10.99	11.44	13.79	9.73	11.73	26.36	29.53	28.28	31.67	3	3	3	3
LAOP	11.26	12.58	9.80	10.93	12.32	13.75	10.47	11.68	33.04	35.09	35.02	37.2	3	3	2	2
SCOP	11.16	12.51	10.17	11.39	11.57	12.96	10.40	11.65	30.14	32.22	30.85	32.97	3	3	3	3
GALL	10.87	11.87	10.30	11.25	11.32	12.36	10.62	11.59	33.04	34.73	34.19	35.94	3	3	3	3
CTOP	9.32	11.43	8.91	10.77	8.71	10.53	8.21	9.92	28.86	32.2	26.69	29.78	3	3	3	3
MAOB	9.96	10.98	10.38	11.43	9.42	10.38	9.80	10.8	32.92	34.8	31.18	32.96	3	3	3	3
VATB	9.73	10.87	10.12	11.28	9.72	10.84	10.19	11.36	30.36	32.37	30.68	32.71	3	3	3	3
FLUF	9.57	10.75	8.46	9.48	9.64	10.8	8.58	9.62	26.12	27.89	26.44	28.23	3	3	3	3
MNOP	9.25	10.44	8.91	10.02	8.79	9.89	8.47	9.53	30.46	32.5	29.12	31.07	3	3	3	3
MSOP	8.70	10.24	8.83	10.3	9.86	11.51	9.87	11.52	27.42	30.05	30.60	33.52	3	3	3	3
KYDA	8.87	10.02	8.80	9.91	9.37	10.55	9.30	10.47	31.54	33.66	33.25	35.47	3	3	3	3
NYWN	7.37	9.79	7.32	9.42	7.16	9.21	7.29	9.37	23.78	27.55	23.69	27.44	3	3	3	3
NYFL	7.53	9.39	8.25	10.09	7.36	9.01	8.24	10.09	24.37	27.55	24.43	27.62	3	3	3	3
CAOP	8.59	9.24	9.12	9.81	8.15	8.77	8.64	9.29	33.09	34.41	31.41	32.66	3	3	3	3
FLMP	8.14	9.15	8.81	9.88	7.93	8.89	8.61	9.65	26.40	28.25	25.94	27.76	3	3	3	3
NYRT	7.71	8.53	8.80	9.71	7.62	8.4	8.74	9.64	26.68	28.29	26.68	28.29	3	3	3	3
TXSA	7.53	8.5	7.46	8.38	7.96	8.94	7.86	8.83	24.86	26.54	26.35	28.13	3	3	3	3
AROR	6.10	7.5	6.72	8.12	6.64	8.02	7.37	8.9	24.13	26.81	26.27	29.2	3	3	3	3
TNMS	5.86	7.34	5.43	6.66	6.57	8.06	5.98	7.33	20.20	22.53	21.89	24.42	3	3	3	3

ADI: area deprivation index; CALC: cause, age, location, consistent; CI: confidence interval; OPO: organ procurement organization

[a] Excludes HIOP which can be seen on Supplemental Table 4

[b] 95% upper confidence limit

[c] Uses unadjusted donation rate and age-adjusted transplant rate.

[d] Uses age-adjusted donation rate and age-adjusted transplant rate.

[d] Uses ADI-adjusted donation rate and age, ADI-adjusted transplant rate.

[f] Uses Age, ADI-adjusted donation rate and age, ADI-adjusted transplant rate.

© 2023 Lopez R et al. *JAMA Network Open.*

eTable 4. Donation rates, kidney transplant rates and tier assignments for HIOP, 2018-2020

Year	Donation rates								Kidney transplant rates								Tiers			
	CALC (unadjusted)		Age-adjusted		ADI-adjusted		Age, ADI-adjusted		CALC (unadjusted)		Age-adjusted		ADI-adjusted		Age, ADI-adjusted		CALC tier[b]	Age-adjusted tier[c]	ADI-adjusted tier[d]	Age, ADI-adjusted tier[e]
	Rate	95% CI[a]	Rate	95% CI[a]	Rate	95% CI[a]	Rate	95% CI[a]	Rate	95% CI[a]	Rate	95% CI[a]	Rate	95% CI[a]	Rate	95% CI[a]				
2018	8.35	10.82	9.03	11.41	7.62	9.62	8.06	10.19	14.99	18.02	16.57	19.83	13.32	15.93	14.36	17.19	2	1	2	2
2019	7.41	9.72	7.40	9.43	6.95	8.85	6.84	8.71	13.17	16.01	13.21	15.92	12.04	14.52	11.87	14.31	3	3	3	3
2020	10.39	13.37	11.48	14.47	9.71	12.23	10.62	13.38	18.70	22.32	21.35	25.49	17.12	20.44	19.25	22.98	1	1	2	1

ADI: area deprivation index; CALC: cause, age, location, consistent; CI: confidence interval; OPO: organ procurement organization

[a] 95% upper confidence limit

[b] Uses unadjusted donation rate and unadjusted kidney transplant rate.

[c] Uses age-adjusted donation rate and age-adjusted kidney transplant rate.

[d] Uses ADI-adjusted donation rate and ADI-adjusted kidney transplant rate.

[e] Uses Age, ADI-adjusted donation rate and age and ADI-adjusted kidney transplant rate.

© 2023 Lopez R et al. *JAMA Network Open.*

eTable 5. Threshold values used for tier assignments

	2018		2019		2020	
	Top 25%	Median	Top 25%	Median	Top 25%	Median
**CALC tier thresholds**						
**Unadjusted donation rate**	11.42	9.58	11.88	10.09	13.14	10.99
**Age-adjusted transplant rate**	36.52	32.04	38.65	32.35	40.55	35.88
**Unadjusted kidney transplant rate for HIOP**	17.82	14.80	18.20	15.22	20.45	17.59
**Age-adjusted tier thresholds**						
**Age-adjusted donation rate**	11.12	9.93	11.16	10.19	12.72	11.05
**Age-adjusted transplant rate**	36.52	32.04	38.65	32.35	40.55	35.88
**Age-adjusted kidney transplant rate for HIOP**	17.16	15.77	18.14	16.02	20.52	17.89
**ADI-adjusted tier thresholds**						
**ADI-adjusted donation rate**	11.40	9.47	11.74	9.94	12.67	11.06
**Age and ADI-adjusted transplant rate**	37.73	32.01	38.03	33.08	40.13	36.30
**ADI-adjusted kidney transplant rate for HIOP**	17.31	14.99	18.63	15.28	20.53	17.65
**Age and ADI-adjusted tier thresholds**						
**Age and ADI-adjusted donation rate**	11.39	9.65	11.42	10.25	12.41	11.12
**Age and ADI-adjusted transplant rate**	37.73	32.01	38.03	33.08	40.13	36.30
**Age and ADI-adjusted kidney transplant rate for HIOP**	17.10	15.49	17.94	15.55	19.76	18.18

Threshold values are calculated using all 58 OPOs

ADI: area deprivation index; CALC: cause, age, and location consistent; OPO: organ procurement organization

© 2023 Lopez R et al. *JAMA Network Open.*

eTable 6. List of Organ Procurement Organization (OPO) Codes and Names

OPO Code	OPO Name
ALOB	Legacy of Hope
AROR	Arkansas Regional Organ Recovery Agency
AZOB	Donor Network of Arizona
CADN	Donor Network West
CAGS	Sierra Donor Services
CAOP	OneLegacy
CASD	Lifesharing - A Donate Life Organization
CORS	Donor Alliance
CTOP	LifeChoice Donor Services
DCTC	Washington Regional Transplant Community
FLFH	OurLegacy
FLMP	Life Alliance Organ Recovery Agency
FLUF	LifeQuest Organ Recovery Services
FLWC	LifeLink of Florida
GALL	LifeLink of Georgia
HIOP	Organ Donor Center of Hawaii
IAOP	Iowa Donor Network
ILIP	Gift of Hope Organ & Tissue Donor Network
INOP	Indiana Donor Network
KYDA	Kentucky Organ Donor Affiliates
LAOP	Louisiana Organ Procurement Agency
MAOB	New England Organ Bank
MDPC	The Living Legacy Foundation of Maryland
MIOP	Gift of Life Michigan
MNOP	LifeSource Upper Midwest Organ Procurement Organization
MOMA	Mid-America Transplant Services
MSOP	Mississippi Organ Recovery Agency
MWOB	Midwest Transplant Network
NCCM	Lifeshare of the Carolinas
NCNC	HonorBridge
NEOR	Live On Nebraska
NJTO	New Jersey Organ and Tissue Sharing Network OPO
NMOP	New Mexico Donor Services
NVLV	Nevada Donor Network
NYAP	Center for Donation and Transplant
NYFL	Finger Lakes Donor Recovery Network

© 2023 Lopez R et al. *JAMA Network Open.*

NYRT	LiveOnNY
NYWN	Upstate New York Transplant Services Inc
OHLB	LifeBanc
OHLC	Life Connection of Ohio
OHLP	Lifeline of Ohio
OHOV	LifeCenter Organ Donor Network
OKOP	LifeShare Transplant Donor Services of Oklahoma
ORUO	Pacific Northwest Transplant Bank
PADV	Gift of Life Donor Program
PATF	Center for Organ Recovery and Education
PRLL	LifeLink of Puerto Rico
SCOP	We Are Sharing Hope SC
TNDS	Tennessee Donor Services
TNMS	Mid-South Transplant Foundation
TXGC	LifeGift Organ Donation Center
TXSA	Texas Organ Sharing Alliance
TXSB	Southwest Transplant Alliance
UTOP	DonorConnect
VATB	LifeNet Health
WALC	LifeCenter Northwest Organ Donation Network
WIDN	Versiti Wisconsin, Inc
WIUW	UW Health Organ and Tissue Donation

© 2023 Lopez R et al. *JAMA Network Open.*

eTable 7. Reclassification rates as compared to CALC rankings[a] for donation and transplant rates

Adjustment	2018	2019	2020
	n (%) out of 58 OPOs		
Donation Rate			
Age-adjusted donation ranking [b]	12 (20.7)	13 (22.4)	18 (31.0)
ADI-adjusted donation ranking [b]	11 (19.0)	9 (15.5)	14 (24.1)
Age, ADI-adjusted donation ranking [b]	12 (20.7)	13 (22.4)	17 (29.3)
Organ Transplant Rate			
ADI-adjusted transplant ranking [b,c]	20 (34.5)	10 (17.2)	8 (13.8)
Age, ADI-adjusted transplant ranking [b,d]	19 (32.8)	10 (17.2)	7 (12.1)

ADI: area deprivation index; CALC: cause, age, location, consistent; CMS: Centers for Medicare & Medicaid Services.

[a] CMS CALC ranking for donation rate is unadjusted. CMS CALC ranking for transplant rates is based on age-adjusted transplant rate for all OPOs except HIOP; the raw kidney transplant rate is used for HIOP.

[b] Rankings for donation and transplant are defined as follows:

Rank 1: OPOs have the upper 95% confidence limit at or above the top 25 percent cutoff for the corresponding rate

Rank 2: OPOs have the corresponding upper 95% confidence limit below the 25 percent cutoff

Rank 3: OPOs have the corresponding upper 95% confidence limit below the median cutoff

[c] Both transplant rates (for OPOs other than HIOP) and kidney transplant rate (for HIOP) are ADI-adjusted.

[d] Both transplant rates (for OPOs other than HIOP) and kidney transplant rate (for HIOP) are age, ADI-adjusted.

© 2023 Lopez R et al. *JAMA Network Open.*
