# Supplementary material for: Population Characteristics and Organ Procurement Organization Performance Metrics
Source: JAMA Netw Open. 2023 Oct 3;6(10):e2336749. doi: 10.1001/jamanetworkopen.2023.36749 (PMC10548299; doi:10.1001/jamanetworkopen.2023.36749)
Supplement: Supplement 2. — Data Sharing Statement [file jamanetwopen-e2336749-s002.pdf]

Data Sharing Statement

Lopez. Population Characteristics and Organ Procurement Organization Performance Metrics. *JAMA Netw Open*. Published October 03, 2023. doi:10.1001/jamanetworkopen.2023.36749

Data
Data available: Yes
Data types: Other (please specify)
Additional Information: Data that is publically available
How to access data: The data derives in part from publically available data. The other data is purchased research data from ahrq and would only be available through a data use agreement.
When available: With publication

Supporting Documents
Document types: None

Additional Information
Who can access the data: Researchers with approved research.
Types of analyses: Any purpose.
Mechanisms of data availability: Approved proposal.
